# Protein Nutrition and Malnutrition in CKD and ESRD

**DOI:** 10.3390/nu9030208

**Published:** 2017-02-27

**Authors:** Yan Zha, Qi Qian

**Affiliations:** 1 Department of Nephrology, Guizhou Provincial People’s Hospital, Guizhou 550002, China; zhayan72@126.com; 2 Division of Nephrology and Hypertension, Department of Medicine, Mayo Clinic College of Medicine, 200 First Street SW, Rochester, MN 55905, USA

**Keywords:** protein nutrition, protein catabolism, chronic kidney disease, dialysis, acidosis, inflammation, hormonal derangements, uremic toxins

## Abstract

Elevated protein catabolism and protein malnutrition are common in patients with chronic kidney disease (CKD) and end-stage renal disease (ESRD). The underlying etiology includes, but is not limited to, metabolic acidosis intestinal dysbiosis; systemic inflammation with activation of complements, endothelin-1 and renin-angiotensin-aldosterone (RAAS) axis; anabolic hormone resistance; energy expenditure elevation; and uremic toxin accumulation. All of these derangements can further worsen kidney function, leading to poor patient outcomes. Many of these CKD-related derangements can be prevented and substantially reversed, representing an area of great potential to improve CKD and ESRD care. This review integrates known information and recent advances in the area of protein nutrition and malnutrition in CKD and ESRD. Management recommendations are summarized. Thorough understanding the pathogenesis and etiology of protein malnutrition in CKD and ESRD patients will undoubtedly facilitate the design and development of more effective strategies to optimize protein nutrition and improve outcomes.

## 1. Introduction

Chronic kidney disease (CKD) has become a worldwide epidemic with an occurrence rate in the population of approximately 5%–15% [1]. Prevalence of end-stage renal disease (ESRD) population relying on dialysis is also on the rise [2]. Suboptimal nutritional intake is common in the population of CKD and ESRD and poses a direct risk for protein malnutrition [3,4]. Suboptimal nutritional status has been related to multiple alterations including metabolic acidosis, bowel flora alteration and hormonal dysregulation, all of which could promote kidney disease progression and increase morbidity and mortality. This review presents updated information, intergrading previous knowledge with a specific focus on the unique aspect of protein balance and nutrition in CKD and ESRD. Current practice recommendations are presented.

## 2. Protein Nutrition in Healthy Adults and in CKD and ESRD

The USA Food and Nutrition Board of the National Academy of Sciences suggests that a minimum dietary protein requirement for a healthy adult in a stable non-pregnant, non-lactating and non-recovery condition is 0.6 g/kg/day. Considering a safety margin, the “Recommended Dietary Allowance” (RDA) of protein intake is 0.8 g/kg/day [5]. It is recommended that greater than half of the protein intake should be of a high biologic value (HBV, nitrogen incorporated into the body/total absorbed nitrogen >75%), such as proteins in eggs, fish, poultry, meat and dairy products. The key feature of HBV proteins is the presence of essential amino acids (the amino acids that are not produced by the body and are required from dietary intake). Studies have shown, however, that the daily protein consumption of an average American is approximately 1.3 g/kg/day, 1.25 and 1.36 g/kg/day for women and men, respectively. Even among individuals over age 75, daily protein consumption remains at approximately 1.1 g/kg/day, considerably higher than recommended intake [6].

Dietary proteins are digested to amino acids which can be further broken down to generate both acids and bases. Proteins from meat and dairy products (from a typical Western diet) generate predominantly acidic products including hydrogen chloride (HCl), sulfuric acid (H_2_SO_4_) and phosphoric acids (H_3_PO_4_). These acids are nonvolatile and rely on kidney for their excretion (primarily in the form of ammonium salts and phosphoric salts). A healthy individual generates net acids, approximately 1 mEq/kg/day (mmol/kg/day), referred to as NEAP (net endogenous acid production). These are rapidly buffered by sodium bicarbonate (NaHCO_3_) to form sodium salts. During this process, bicarbonate is consumed, which needs to be regenerated, a task accomplished by the kidneys. To achieve a steady acid–base balance, renal tubules must reabsorb ~4500 mEq of filtered HCO_3_^−^ and generate (through H^+^ excretion) an additional ~70–80 mEq HCO_3_^−^ daily, to neutralize the daily net acid generation [7]. In patients with reduced kidney function, nonvolatile acids can accumulate causing metabolic acidosis.

## 3. Metabolic and Regulatory Derangements in CKD and ESRD

**Figure 1:**
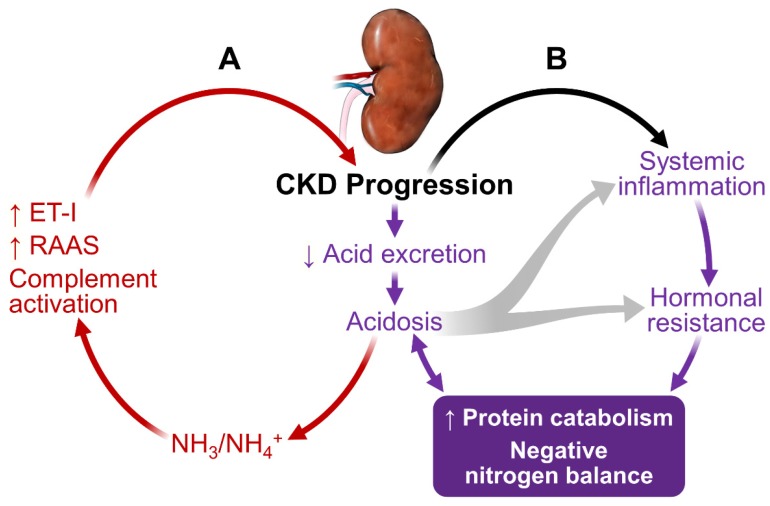
Interconnection of CKD progression with metabolic acidosis, inflammation, hormonal resistance and protein catabolism. (**A**) Kidney dysfunction limits proton (H^+^) excretion, resulting in a systemic metabolic acidosis. The acidosis causes activation of complement systems, renin angiotensin aldosterone systems and endothelin-1. These acidosis-mediated effects cause CKD progression, forming a viscous cycle; (**B**) Acidosis promotes inflammation and tissue resistance to multiple anabolic hormones and simultaneously enhances activity of catabolic corticosteroids. Protein catabolism generates acidic products, contributing to acidosis in the setting of CKD and ESRD. Collectively, these abnormalities give rise to a state of protein catabolism, causing sustained negative nitrogen balance, leading to muscle wasting.

As shown in Figure 1, kidney dysfunction is associated with defects in acid excretion, systemic inflammation, end-organ hormone resistance and uremic toxin accumulation. These abnormalities can further worsen kidney function, creating a vicious circle, adversely affect patients’ outcome.

### 3.1. Metabolic Acidosis

In CKD, the reduced number of functioning nephrons compromises the kidney’s capacity to excrete acid [8]. Metabolic acidosis is the earliest and one of the most common manifestations of CKD. The acidosis worsens progressively as CKD progresses [9,10,11,12]. At the individual nephron level, each residual functioning nephron undergoes compensatory hypertrophy and generates an excess amount of NH_3_ in an attempt to excrete acid in the form of NH_4_^+^ [13,14]. NH_3_/NH_4_^+^ can lead to complement activation, resulting in tubule-interstitial inflammation, injury and fibrosis [15]. Acidosis also increases endothelin-1 and aldosterone production, furthering CKD progression [16,17].

Metabolic acidosis plays an important role in the accelerated protein catabolism, negative nitrogen balance and loss of lean body mass in CKD and ESRD [18,19]. Acidosis activates proteolysis through activating the ubiquitin-proteasome system (UPS) and caspase-3 [20]. Caspase-3 cleaves actomyosin and myofibrils, providing suitable substrates for UPS-mediated degradation. Caspase-3 also cleaves subunits (Rpt2 and 6) of 19S proteosome particle to activate the 26S proteosome-mediated protein degradation. Thus, acidosis in CKD can preferentially cause muscle protein breakdown to a much greater extent than mobilizing protein from other organs. Acidosis also contributes to insulin resistance, growth hormone resistance and glucocorticoid hypersecretion. These hormonal defects contribute to the protein catabolic state (detailed below).

It is not surprising that acidosis promotes CKD progression [21] and increases mortality [22]. Importantly, a normal range of serum HCO_3_^−^ concentrations is associated with favorable clinical outcomes [23,24] and correction of acidosis corrects virtually all known adverse effects associated with acidosis in CKD and ESRD [15,25,26,27,28].

### 3.2. Sustained Inflammation

**Figure 2:**
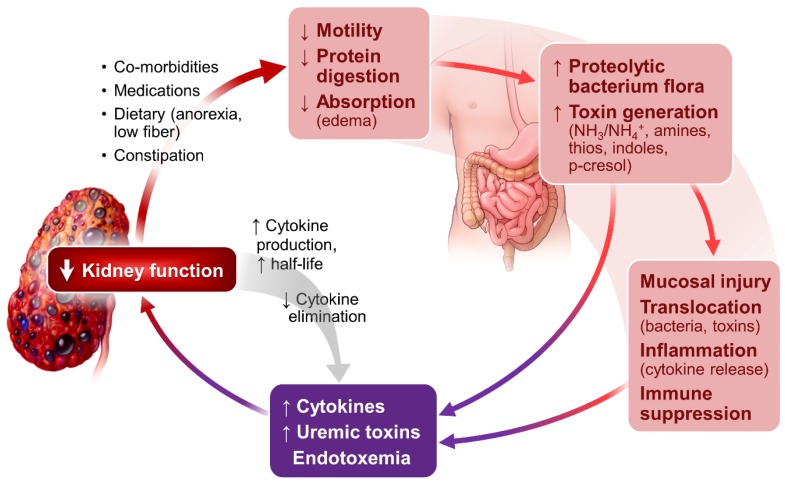
Kidney-intestinal axis: Gastrointestinal-related inflammation and uremic toxin generation in CKD and ESRD. With progressive decline in kidney function, cytokine production and half-life increases while cytokine elimination decreases. CKD patients frequently have comorbidities (diabetes, hypertension, cardiovascular diseases, anemia and hyperphosphatemia) and are on multiple medications including diuretics (often with fluids restriction), iron preparations, phosphate binders and more that can slow bowel transit and cause constipation. With imposed dietary restrictions for CKD and ESRD, they tend to be on a diet low in fiber that can affect gut motility. Slow intestinal transit plus bowel mucosa edema (occurs often when patients with worsening kidney dysfunction and cardiovascular abnormalities) could alter protein digestion and bowel flora. Proteolytic bacteria floras are preferentially selected over the saccarolytic bacteria. The predominant proteolytic bacteria ferment luminal proteins and generate toxic metabolites and ammonia. The toxic metabolites would disrupt intestinal mucosal barriers causing increasing bacteria and toxin translocation. The systemic exposure and accumulation of these toxins and bacteria causes a low-grade but persistent endotoxemia. Adequate gut flora is known to enhance bowel integrity and promote immunoregulatory function. Disruption of normal flora could compromise gut immune function and, in the extreme, create a situation of “immunoparalysis”. The consequence of these effects is systemic cytokine activation, accumulation of uremic toxins and endotoxemia (purple rectangle).

Sustained systemic and tissue inflammation is a prominent feature of CKD and ESRD [29,30]. It is related to a multitude of abnormalities in the setting of kidney failure. As illustrated in Figure 2, altered gut microbiome profile, evidenced in even the early stages of CKD [31] and in ESRD [32,33], plays an important role in the genesis of inflammation. Impaired protein digestion [34] increases intestinal protein fermentation by proteolytic bacteria and increases production of toxic metabolites including NH_3_/NH_4_OH, which are injurious to intestinal mucosa [35,36,37].

The intestinal epithelial injury results in the translocation of toxic metabolites and bacteria endotoxins from intestinal lumen to the circulation, stimulating production of inflammatory cytokines through binding the lipopolysaccharides and Toll-like receptors [38,39]. Impaired kidney elimination of uric acid also fosters the selection of gut bacteria that produce urease and uricase. Most of these bacteria are capable of generating toxins including indole and p-cresol, contributing to uremia in the setting of CKD and ESRD [40,41].

The generation and systemic accumulation of uremic toxins exemplify the importance of gut flora in the pathogenesis of uremic syndrome. Gut-derived indole and p-cresol are sulfonated in the liver, forming uremic toxins, indoxyl sulfate (IS) [42] and p-Cresyl sulfate (pCS). IS mediates renal tubulointerstitial fibrosis via upregulation and activation of TGF-β1 [43]. pCS increase the production of inflammatory cytokines and oxidative stress in CKD [44]. A recent in vitro study show that pCS induces macrophage activation but interfere with antigen processing, likely contributing to a compromised adaptive immune response [45]. Both IS and pCS activate RAAS, in additional to activating TGF/Smad pathways, and likely contribute to epithelial–mesangial transformation and CKD progression [46]. IS and pCS are associated with increased cardiovascular and all-cause mortality in patients with CKD and ESRD [47,48,49,50,51]. The readers are referred to several excellent comprehensive updates on uremic toxins [52,53] and the contribution of intestinal bacteria in the generation of uremic toxins and systemic inflammation [54].

In addition to gut source of inflammation, fat distribution is also associated with inflammatory state. Studies have shown that abdominal fat, not peripheral fat, is highly associated with inflammation, insulin resistance, dyslipidemia, and oxidative stress as well as cardiovascular events in CKD [55,56,57,58,59,60,61]. Dialysis and dialysis-related factors, both hemodialysis and peritoneal dialysis, are also a prominent source of inflammation [30,62,63].

Negative protein balance in the inflammatory state in CKD and ESRD can be ascribed to the activation of multiple cytokine (TNF, IL-1 and IL-6)-mediated mechanisms [64,65,66]. For instance, TWEAK (TNF-related weak inducer of apoptosis) is associated with pathways in the regulation of NF-kappa B (nuclear factor kappa light chain enhancer of activated B cells). It inhibits myogenesis and activates muscle protein degradative cascades [67]. TWEAK, as well as IL-6, is highly expressed in patients with kidney failure, and both are shown to be associated with reduced muscle strength in dialysis patients [68]. Myostatin, a TNF-beta superfamily protein, is upregulated in CKD and can be further activated by free radicals. Myostatin binds to muscle membrane ActRIIB (high-affinity type 2 Activin receptor), activates ALK-4 and ALK-5 (type I activin receptor serine kinases). These kinases trigger downstream phosphorylation of Smads 2 and 3, leading to signal activations that ultimately result in muscle degradation and atrophy. These effects are confirmed by multiple experimental loss-of-function myostatin mutations or deletions in animal models which consistently show that attenuation of myostatin leads to an increase in the size and number of skeletal muscle fibers and muscle mass [69,70], opposite to the observations in CKD and ESRD. Overexpression of an endogenous myostatin inhibitor (follistatin isoform) increases muscle mass and strength. Commensurate with gene manipulating studies, muscle levels of myostatin in the aging population and CKD patients are shown to be elevated [70], contributing to a negative muscle protein balance. Inhibiting cytokine pathways of myostatin in CKD can mitigate inflammation-associated muscle protein degradation, improve sensitivity to insulin/IGF-1 and reduce muscle protein breakdown, leading to increased muscle growth [71,72]. Moreover, exercise upregulates follistatin and the increase is associated with increased muscle strength and mass in patients with CKD [73,74].

Inflammation also induces multiple hormonal derangements including enhancing glucocorticoid-mediated effects and mitigating insulin/IGF-1 effects by inducing tissue resistance [75]. Muscle protein degrades, in part, through activation of intracellular NADPH oxidases [76,77,78]. IL-6 has also been shown to interact with serum amyloid A leading to impairment of insulin-IGF-1 signaling via activation of suppressor of cytokine signaling (SOCS3) and downstream loss of insulin receptor substrate 1 (IRS-1) in muscle [79]. Moreover, IL-6 mediated signaling impairs assimilation of endogenous amino acids for muscle protein synthesis and enhanced caspase-3 activity, further compromising protein nitrogen and muscle protein balance [80,81].

Collectively, inflammation, through a complex array of mechanisms, preferentially increases in muscle protein catabolism and suppresses muscle protein anabolism, leading to a net muscle protein loss in CKD and ESRD.

### 3.3. Hormonal Disorders

Hormonal disorders are prominent in CKD [82]. Acidosis, inflammation and uremic toxins have all been shown to contribute to the hormonal dysregulation in CKD and ESRD. Strong evidence demonstrates the existence of tissue resistance to insulin, growth hormone-insulin-like growth factor-insulin-like-growth factor binding protein (GH-IGF-IGFBP) axis, gonadal hormone (testosterone) and vitamin D. Catabolic activity of glucocorticoids, however, is elevated.

In CKD and ESRD, insulin resistance is associated with significantly elevated protein catabolism, due primarily to post-receptor defects and activation of UPS, leading to muscle protein degradation rather than reduced hepatic glucose intake. Importantly, the insulin resistance-related muscle protein breakdown in dialysis patients is seen not only in patients with diabetes but also in dialysis patients without overt diabetes. Notably, the negative nitrogen balance and hyperaminoacidemia resulting from elevated protein breakdown can be reversed through insulin administration [83]. Moreover, dialysis can correct, at least in part, diminished insulin response and improve tissue insulin sensitivity [84,85,86,87].

The GH-IGF-IGFBP axis plays an important role in kidney development and kidney diseases. Adequate activity of the axis enhances renal blood flow and GFR and can cause Na^+^ retention-mediated volume expansion [88]. CKD is associated with multiple derangements in the GH-IGF-IGFBP axis [89,90,91]. Although the plasma GH levels may be normal or elevated in CKD [90] due to limited GH clearance, at the tissue level, there is evidence of GH resistance, leading to insufficient downstream effects [92,93]. Serum IGF-1 levels may also be normal (or reduced in advanced CKD). Its circulating binding proteins, IGFBPs, tend to be elevated due to a heightened hepatic production and diminished renal clearance. IGFBPs bind to circulating IGF-1, which in most instances results in a decreased IGF bioavailability [94,95]. Tissue resistance to IGF-mediated effects has also been demonstrated in uremic rodents [96]. Urinary loss of IGF and IGFBP can also be significant in patients with nephrotic syndrome and contributes to the inadequate IGF-related functions [97].

Given the tissue resistance to GH and IGF-1, it should come as no surprise that children with kidney failure exhibit growth retardation. In adults, such hormone resistance manifests as an accelerated protein catabolism and protein malnutrition. Altered PI_3_-kinase/AKT activity, downstream signaling of growth factors (insulin and IGF-1), activates caspase-3 and enhances cleavage of actomyosin complexes and myofibrils [98] and is a critical step in muscle protein degradation. The signaling impairment also alters muscle satellite cell function [99], interfering with muscle injury repair and maintenance of muscle mass [79]. All of these degradative processes can be exacerbated in the context of insufficient energy provision. Thus, GH-IGF-IGFBP axis dysfunction plays an important role not only in growth but also in nitrogen and muscle protein balance.

Emerging studies have uncovered a novel regulatory signaling pathway of insulin/IGF mediated by a number of muscle specific micro-RNAs [100,101,102]. In CKD muscle, the microRNA expression pattern is altered [71]. For instance, mir-29 in CKD muscle is depressed, increasing YinYang1 protein and negatively regulating myogenesis [71]. Further studies in this area are necessary to improve our understanding of the complex mechanisms underlying muscle protein loss in CKD and ESRD. Manipulating muscle specific miroRNAs could constitute potentially useful novel targets for interventions to prevent and treat muscle protein loss and protein malnutrition.

Testosterone, a prototypical anabolic hormone, induces skeletal muscle hypertrophy and positive nitrogen balance under physiological conditions. Testosterone also inhibits expression of myostatin-mediated muscle protein degradation, induces muscle response to IGFs, promotes IGF-1 mRNA expression and recruits pluripotent stem cell differentiation into myocytes [103]. CKD patients have a blunted circulating testosterone level. This is due primarily to prolactin accumulation in CKD, leading to impaired gonadotropin releasing hormone secretion from the anterior pituitary, which, in turn, causes testosterone deficiency [104,105]. Dramatic reversal of this otherwise nearly universal occurrence of hypogonadism in dialysis patients after successful kidney transplantation [106,107] illustrates the highly toxic but reversible nature of the uremic milieu. Even at the early stages of CKD, a significant association of muscle mass loss with reduced endogenous testosterone has been demonstrated [108], a major component of negative protein balance patients with renal failure.

Another notable hormonal defect in CKD and ESRD is the heightened production and activity of glucocorticoids. Metabolic acidosis and inflammatory state in CKD pathologically enhances adrenal glucocorticoid production and activity. Glucocorticoids in CKD and ESRD, through activating the glucocorticoid receptor and binding to phosphatidylinositol 3-kinase, suppress Akt phosphorylation [75], a defect further magnified by the parallel presence of acidosis [109]. Reduction in phosphorylated Akt contributes to the muscle protein degradation through multiple mechanisms including upregulating proteolytic pathways and impairment of intracellular growth hormone (insulin/IGF-I)-mediated signaling pathways [98,110,111].

Suboptimal vitamin D status, common in patients with CKD and ESRD [112], has also been associated with muscle protein imbalance and catabolism. Tissue resistance to and reduced circulating 25-OH vitamin D, as well as increased fibroblast growth factor-23, worsens secondary hyperparathyroidism [113], which contributes to muscle degradation. Vitamin D has been shown to be involved in pathways of muscle regulation [114,115,116]. Moreover, replacement of active form of vitamin D (1,25-OH_2_ vitamin D) has been shown to improve muscle size and strength, markers of muscle metabolism as well as serum albumin concentration [115,117]. Vitamin D deficiency in CKD contributes to RAAS hyperactivation, causing multiple detrimental downstream effects including compromised mental status [118]. Altered mental status can lead to poor dietary intake, contributing to poor nutritional intake, intestinal dysmicrobia, accumulation of uremic toxins, and, ultimately, worsening protein malnutrition.

Taken together, in CKD and ESRD, there is enhanced activity of catabolic hormones and reduced activity and resistance to anabolic hormones. These changes together with other uremic conditions including acidemia, inflammation and decreased nutritional intake can work in concert to cause a persistent net negative nitrogen balance and loss of lean body mass.

## 4. Energy Prescription and Protein-Energy Wasting (PEW) in CKD and ESRD

Protein catabolism and nitrogen balance in CKD are tightly linked to energy intake [119]. A negative energy intake accelerates protein catabolism as protein being used from energy supply, leading to a negative nitrogen balance.

Multiple studies have shown that in patients with CKD and ESRD, their resting energy expenditure is increased compared to non-CKD individuals [120,121,122,123,124]. Inflammatory state and co-morbidities associated with CKD and ESRD such as cardiovascular disease, poorly controlled diabetes, and hyperparathyroidism can all contribute to the increased resting energy expenditure [121,123,124,125,126,127]. Resting energy expenditure is shown to increase from 12% to 20% during dialysis [128]. Thus, patients with renal failure require a higher amount of energy intake than healthy individuals. CKD and ESRD patients are, thus, susceptible to insufficient energy intake.

In non-dialysis CKD patients, a neutral or slightly positive nitrogen balance can be maintained with a low quantity (~0.6 g/kg/day) but high quality (HBV) protein diet and adequate energy intake (30–35 kcal/kg/day) [129]. With adequate energy intake (ketogenic diet) and supplemental amino acids, even with very low (0.3 g/kg/day) protein intake, CKD patients can maintain a neutral nitrogen balance and stable clinical status [130]. These dietary related favorable effects are allegedly derived from reduced generation of toxic waste products and enhanced insulin/IGF sensitivity. A recent meta-analysis by Jiang [131] adds weight to the existing impression that a low protein diet is effective in management of CKD without necessarily causing adverse safety and nutritional effects. An individualized meal plan should be devised under the supervision of a nephrology dietitian.

In patients on maintenance hemo- or peritoneal dialysis, their protein requirement is, on the contrary, much higher, 1.2–1.3 g/kg/day, based on KDOQI (Kidney Disease Outcomes Quality Initiative) Clinical Practice Guideline for Nutrition. The higher protein requirement is due to dialysis related protein loss, extra-energy expenditure and persistent inflammation [132]. Protein intake at <0.8 g/kg/day or >1.4 g/kg/day has been shown to be associated with increased mortality in dialysis patients [133].

Protein-energy wasting (PEW) denotes concurrent losses of protein and energy stores in patients with kidney dysfunction. It tends to develop and progress with CKD progression [134,135]. In the setting of suboptimal energy supply, CKD and ESRD patients catabolize muscle to provide needed energy, leading to protein malnutrition [136]. Limited physical activity in the setting of enhanced resting energy demand, although tempering the total body energy expenditure [137], would inevitably result in muscle atrophy and generalized deconditioning. The readers of this paper are referred to several detailed reviews on the topic [138,139,140,141].

## 5. Clinical Recommendations

An integrated multidisciplinary approach with targeted and individualized nutritional interventions based on the degree of kidney dysfunction, comorbidity, baseline nutritional status and physical functional capacity is necessary to improve outcomes for CKD and ESRD patients. Conditions that contribute to protein catabolism should be minimized or eliminated.

### 5.1. Optimizing Nutritional Therapy

Although randomized interventional patient-based trials are scarce, the general consensus is that active nutritional measures can mitigate a large number of the metabolic and hormonal derangements in CKD and ESRD. For patients with relatively stable health conditions, absence of active medical events and not on dialysis, nutritional assessment including intake and anthropometric measurements (body weight, BMI and valid indicators of nutritional status [142]) every 3–6 months is advisable. For patients with active medical issues such as surgical operation, acute infection, or cardiovascular events, more frequent nutritional analysis and modifications are required. Ongoing modification of nutritional parameters is necessary. Once a patient reaches ESRD, monthly review of the patient’s laboratory data and dietary intake by a renal dietitian or a nutritional professional is advised.

Dietary energy provision for both dialysis and non-dialysis CKD patients should be 30–35 kcal/kg (ideal body weight)/day. The recommended amount of protein intake for non-dialysis CKD patients is 0.6 to 0.8 g/kg/day with >50% HBV proteins. For patients on peritoneal dialysis and hemodialysis, dietary protein intake in the range of 1.0–1.2 g/kg/day is advised.

With a low protein and adequate energy diet, hyperphosphatemia can often be minimized in non-dialysis CKD patients. In dialysis patients requiring high protein intake, risk for hyperphosphatemia can be substantial and should be carefully monitored and when appropriate, phosphate binders should be promptly instituted [143]. A recent prospective study by Rhee et al. of a cohort (*n* = 110) of hypoalbuminemic hemodialysis patients showed beneficial effects of increasing serum albumin (≥0.2 mg/dL) and maintaining serum phosphorous levels within a target range (3.5 to <5.5 mg/dL) by providing high-protein meals during hemodialysis combined with lanthanum carbonate administration [144]. It should be noted, however, the occurrence of intradialytic hypotension associated with the feeding was not detailed. In patients with intercurrent acute illness that causes hypercatabolism, temporarily enhancing protein intake may be necessary to meet the demands. Protein, amino acids and ketoacid supplementation may be effective in improving protein energy wasting irrespective of the etiology [145]. It remains, however, to be tested as to whether such supplementation can translate to better clinical outcomes such as survival or reduced CKD progression.

### 5.2. Correcting Metabolic Acidosis

Metabolic acidosis should be corrected with sodium bicarbonate (NaHCO_3_). NaHCO_3_ corrects acidosis in children with renal tubular acidosis and stimulates growth in premature infants and children with kidney failure. NaHCO_3_ and potassium bicarbonate improve nitrogen balance in elderly with even mild metabolic acidosis [146,147]. Based on available evidence and while awaiting results from several larger sized randomized interventional trials [148,149,150], we suggest (1) increasing dietary alkali (fruits and vegetables) for patients in stages 3 and 4 CKD and with preserved NaHCO_3_ (22*–*24 mmol/L) [150], and (2) initiating oral NaHCO_3_ for patients with CKD and serum HCO_3_^−^ < 22 mmol/L [151]. The HCO_3_^−^ goal should be 24*–*26 mmol/L. Over correction of HCO_3_^−^ to >26 mmol/L should be avoided [152,153,154].

In dialysis patients (both hemodialysis and peritoneal dialysis), correction of metabolic acidosis reduces protein degradation and negative nitrogen balance [25,155,156,157,158], and significantly improves virtually all hormonal alterations [159], signifying the importance of close monitoring and management of a patient acid–base status as it closely relates to the morbidity and mortality. Over and rapid correction could, however, be detrimental and should therefore be avoided [160]. Graded dialysate HCO_3_^−^ concentrations during each dialysis might minimize the large acid–base fluctuation. Appropriately designed trials are needed to test this assumption.

### 5.3. Eliminating Correctible Inflammatory Factors

Dietary modifications including protein restrictions play a major role in minimizing inflammation in non-dialysis patients. Limiting protein waste products can lead to less uremic toxin elaboration and less toxin-induced inflammation. The positive effects of moderate dietary protein restriction on inflammatory state (IL-6) have been shown in proteinuric diabetic patients [161]. The intestinal source of inflammation can be minimized by preventing dysbiosis through increasing dietary fiber, appropriate treatment of constipation in addition to an adequate dietary provision of protein and energy. The use of probiotics and/or prebiotics is controversial and needs further study. Given the lack of substantial adverse effects, however, these supplementations could be considered when appropriate. Fecal transplantation is theoretically plausible as it might reestablish normal intestinal flora to minimize intestinal source of toxins and inflammation. It, however, needs study in CKD and ESRD population before it can be incorporated into clinical practice.

Volume overload compromises renal perfusion [162] and can worsen the intestinal translocation of endotoxin, bacteria and uremic toxins, contributing to CKD progression [163]. Volume overload should, therefore, be avoided and promptly corrected.

### 5.4. Minimizing Hormonal Alterations

Dietary modification, correcting acidosis and normalizing intestinal flora could all contribute to minimizing hormonal abnormalities seen in CKD and ESRD. In addition, insulin sensitizers (such as metformin, rosiglitazone and pioglitazone) used when appropriate and in patients with mild-to-moderate CKD (within CKD stage 3a, estimated glomerular filtration rate > 45 mL/min/1.73 m^2^), can be administered in conjunction with other diabetes treatment. Muscle protein may be preserved with heightened insulin sensitivity. For CKD patients with hypovitaminosis D, current KQIGO recommendation is to correct the circulating 25-hydroxyvitamin D to an adequate level (>30 ng/mL), which, in general, is the same as that for the general population [118]. It is, however, important to keep in mind that evidence supporting the recommendation is limited. Although correcting hypovitaminosis D has its intuitive appeal, controversy exists [164]. Ongoing clinical follow up and balance pros and cons of the vitamin D supplement are required. Further detailed, carefully designed longer-term interventional studies are needed.

Growth hormone and testosterone use in adult CKD and ESRD is controversial. GH has been used in children with renal failure to foster growth. In adult patients, several studies of GH (and IGF-1) administration in the last decade have been shown to reduce inflammation and muscle catabolism and improve nutritional status in ESRD patients [165,166,167]. Its use, however, has not been incorporated into routine practice. Similarly, several randomized interventional studies using androgen in ESRD patients have shown improvement in muscle mass and nutritional status [168,169,170]. Caution should be exercised, however, as testosterone treatment has been reported to induce a number of treatment-related complications [171]. Moreover, the precise formulation, strength and dosing intervals of anabolic hormones in patients with CKD and ESRD have not been established. Until further studies demonstrate consistent benefit and safety, anabolic hormone supplementation may not be used as a routine treatment modality for adult CKD and ESRD patients.

### 5.5. Increasing Physical Activity

After reaching appropriate amount of protein and energy intake, CKD and ESRD patients should be encouraged to be physically active. Exercise increases expression of the anti-inflammatory protein follistatin, improves sensitivity to IGFs and enhances muscle fiber generation [73,74]. In line with these observations, exercise in rodent CKD models and in limited studies of both pre-dialysis and dialysis patients has been shown to reduce and prevent muscle loss [172,173,174,175]. Although presumed beneficial effects of physical exercise are multiple, appropriate-sized interventional trials are lacking. In practice, physical activity is encouraged in general to preserve lean body mass and maintain protein nutrition balance.

## 6. Summary

In patients with CKD and ESRD, prominent metabolic and regulatory derangements occur including acidosis, systemic inflammation, and hormonal dysregulation that have been attributed to the development of hypercatabolism and risk for negative nitrogen balance. Worse yet, with often concurrent comorbidity and imposed dietary restriction and medications, CKD and ESRD patients commonly experience decreased appetite, anorexia, and a variety of gastrointestinal abnormalities including gastroparesis, slow intestinal transit, diarrhea/constipation and increased gut mucosal permeability. They are at a high risk for developing intestinal dysbiosis and increased intestinal bacteria derived cytokine and uremic toxins. If energy supply is less than optimal, an accelerated loss of lean body mass due to protein degradation will inevitably ensue, leading to increased morbidity and mortality. Existing evidence, although limited by sample size, often retrospective design and secondary analysis of clinical trials, supports the concept that CKD and dialysis patients can benefit from carefully designed nutritional therapy (sufficient energy and an appropriate amount of HBV protein). Further confirmation from prospective randomized controlled trials to examine clinical outcomes such as mortality from nutritional interventions in the CKD and ESRD population is required.

The elucidation of intestinal-kidney bidirectional and dynamic interactions has allowed us to better appreciate the role of diet and nutrition in the pathogenesis of protein energy alterations and maladaptation in CKD and ESRD. Nutritional intervention and manipulation of gut microbiome to obtain a desired array of microbial population in the intestine may represent a novel class of nontoxic and potentially effective strategies to prevent protein malnutrition in CKD and ESRD. Further research to thoroughly understand the complex intestinal-kidney interplay and patient-based trials in this area are needed. Current evidence supports the notion that dietary monitoring and modifications based on the patient clinical condition will likely enhance patient nutritional status and preserve a favorable bowel microbiome, lean body mass and kidney function. Thus, nutritional therapy should be undertaken as one of the critical and renal protective strategies in parallel with other measures.

## Key Points:

1. Protein malnutrition is common in patients with CKD and ESRD, a growing patient population worldwide.
2. Protein malnutrition is associated with increased morbidity and mortality.
3. Protein malnutrition can be prevented and substantially reversed with ongoing dietary monitoring and nutritional therapy.
4. Protein intake of 0.6–0.8 g/kg/day for non-dialysis CKD patients and 1.0–1.2 g/kg/day for patients on peritoneal dialysis or hemodialysis, with >50% HBV proteins, are advised.
5. Daily energy intake in CKD and ESRD patients should be 30–35 kcal/kg (ideal body weight).
6. Metabolic acidosis is related to multiple metabolic derangements, adversely affects kidney and patient outcome, and should be corrected.
7. In patients with stage 3 CKD and without evidence of metabolic acidosis, the dietary modification with increased basis such as vegetables and fruits may be initiated to prevent metabolic acidosis.
8. Constipation and abnormal bowel habits can compromise gut epithelial cell integrity causing dysbiosis, promoting inflammation and uremic toxin accumulation.
9. Exercise, based on patient capacity, should be incorporated in as a part of the CKD and ESRD management.
10. Anabolic hormone replacement is controversial and has not been routinely used for adult CKD and ESRD patients.
11. Current recommendation is to supplement 25(OH)-vitamin D for CKD and ESRD patients with suboptimal vitamin D status.

## Abbreviations

UPS	Proteasome-ubiquitin system
CKD	Chronic kidney disease
ESRD	End-stage renal disease
HBV	High biological value
IS	Indoxyl sulfate
pCS	p-Cresyl sulfate
HCl	Hydrogen chloride
H_2_SO_4_	Sulfuric acid
H_3_PO_4_	Phosphoric acids
NaHCO_3_	Sodium bicarbonate
TWEAK	TNF-related weak inducer of apoptosis
IL-1	Interleukin-1
IL-6	Interleukin-6
NF-kappa B	Nuclear factor kappa light chain enhancer of activated B cells
SOCS3	Suppressor of cytokine signaling
IRS-1	Insulin receptor substrate 1
GH-IGF-IGFBP	Growth factor-insulin-like-growth factor binding protein
IGF-1	Insulin like growth factor-1
KDOQI	Kidney Disease Outcomes Quality Initiative
PEW	Protein-energy wasting
